# Painful Borderline Acetabular Dysplasia: What's New?

**DOI:** 10.1055/s-0044-1790212

**Published:** 2025-04-02

**Authors:** Rodrigo Monari, Fábio Lima Ferreira Pessiquelli, Eduardo Gomes Machado

**Affiliations:** 1 Clínica Monari, Joinville, SC, Brasil; 2 Serviço de Cirurgia do Quadril, Hospital Santo Antônio, Blumenau, SC, Brasil; 3 Instituto Jundiaiense de Ortopedia e Traumatologia, Jundiaí, SP, Brasil

**Keywords:** decision making, diagnostic imaging, femoracetabular impingement, hip dislocation, hip joint

## Abstract

Developmental dysplasia of the hip (DDH) is a complex static-dynamic condition resulting in chronic joint instability and osteoarthritis. Borderline acetabular dysplasia refers to slightly abnormal patterns in the acetabular shape and coverage that are not within the dysplastic range. However, they can predispose to mechanical dysfunction and hip instability. Diagnosis and treatment remain controversial topics in hip preservation, with little current comparative literature to guide accurate diagnosis and treatment decision-making. Historically, the diagnosis of borderline DDH relied on assessments of the acetabular anatomy on anteroposterior pelvic radiography, most commonly the lateral central-edge angle (LCEA), with normal values ranging from 20 to 25° or, in some more recent studies, 18 to 25°. Surgical treatment decision-making debates the use of isolated hip arthroscopy or periacetabular osteotomy, considering the difficulty in determining a fundamental mechanical diagnosis (instability versus femoroacetabular impingement) in subjects with borderline DDH. Therefore, for effective surgical decision-making, the evaluation of additional bone anatomy characteristics, instability, and patients' features is essential.

## Introduction

Developmental dysplasia of the hip (DDH) is a significant musculoskeletal condition. Although around 80% of cases are present from birth, such changes often remain without an appropriate diagnosis. Even though most DDH cases are asymptomatic, the condition can cause biomechanical changes in the hip region, potentially predisposing to the development of hip osteoarthritis. 1

The classification of this condition often relies on the Wiberg lateral center-edge angle (LCEA) values lower than 20°. 2 However, studies show that hips with values between 20 and 25° also present signs of dysplasia but with different imaging, biomechanical, and clinical presentations from established DDH. Thus, we refer to these cases as borderline DDH.

The term “borderline” is somewhat controversial, and its definition in the literature is variable. It is important to note that this term does not indicate that an acetabular deficiency is insignificant but, rather, it recognizes that the primary diagnosis of these hips can fall on either side of the impact and instability spectrum established in the literature. 3 4

One of the major dilemmas related to borderline DDH is that, unlike the established condition, some patients present structural instability (similar to those with established dysplasia), while other patients present femoroacetabular impingement (FAI) and microinstability. 5 6 Therefore, surgical decision-making in borderline acetabular dysplasia is challenging because of the lack of clinical standards to differentiate hips with significant structural instability from those with FAI and microinstability/no instability. 7

Therefore, given the relative scarcity and heterogeneity of the literature regarding borderline DDH, this study aimed to perform a literature review to identify the most current concepts regarding diagnosis, classification, treatment, and clinical outcomes of this condition.

## Methods

### Literature Narrative Review

The article searching and acquisition process relied on a broad literature review in the following databases: Medline (PubMed), OVID, Google Scholar, and Scielo. We created a search strategy for each database to identify articles of interest. Examples of search engines are the following: *borderline hip dysplasia* AND *imaging diagnostic* OR diagnostic OR x-ray OR *magnetic resonance* OR computer tomography or *borderline hip dysplasia* AND *treatment* OR *surgical treatment* OR *hip arthroscopy* OR *periacetabular osteotomy* .

Next, we excluded articles not mentioning hip dysplasia, not written in Portuguese or English, and inaccessible.

## Epidemiology

As it is a borderline condition and there are many debates regarding its classification, the actual prevalence of borderline DDH is not yet completely understood, considering that many studies classify hips with this condition as healthy or do not report their values. Identification can use multiple parameters, including LCEA angles ranging from 20 to 25°, Sharp angles higher than 45°, and Tönnis angles higher than 10°. 5 8

### Epidemiology – General Population

Jacobsen et al. 9 studied 3,859 asymptomatic patients and reported that the prevalence of borderline DDH was 19.2%, while only 3.4% of patients had established DDH.

Furthermore, Engesæter et al., 8 in a prospective study with 2,072 subjects, aged 19-years-old, identified that 16.7% of patients showed signs of borderline DDH and 3.3% had signs of established dysplasia. More recently, a North American study with 2,596 patients collected from a database between 1990 and 1997 to detect the prevalence of osteoarthritis in Johnston County, North Carolina, showed that borderline DDH was around 18.8%. 10

Freiman et al. 11 did a meta-analysis aiming to identify the general prevalence of borderline DDH. These authors reported a rate of 6.7% asymptomatic patients. Furthermore, they pointed out that this condition occurs 3.5 times more often than classic DDH in the general asymptomatic population. However, in symptomatic populations, this difference decreases to 1.3 times.

### Epidemiology – Professional Athlete Population

Kapron et al. 12 analyzed a population of 67 asymptomatic American football players and detected that 19.4% had borderline DDH. A study by the same group with 63 asymptomatic female athletes, with an average age of 19 years, found that 46% of them had borderline and 20% established DDH. 13 Finally, Harris et al. 14 investigated 47 professionals from a ballet company and found that 51% of them had borderline DDH.

### Epidemiology – Symptomatic Population

Regarding the prevalence of borderline DDH in patients with hip pain, Kraeutler et al. 15 studied 341 subjects and diagnosed borderline dysplasia in 14% of them. Matsuda et al. 16 investigated 1,053 patients waiting for hip arthroscopy and identified a prevalence of 12.6%.

## Imaging Diagnosis

The establishment of borderline DDH diagnosis requires imaging tests and the measurement of angles associated with dysplasia-related abnormalities. Table 1 compares the values for established and borderline dysplastic hips.

**Table 1:** Parameters for diagnosing borderline dysplasia or frank dysplasia

Parameter	Borderline dysplasia	Frank dysplasia
LCEA	20–25°	< 20°
Iliofemoral line	15–22%	> 22%
Anterior center-edge angle	20–25°	< 20°
Tönnis angle	> 13°	> 13°
Sharp angle	39–42°	> 42°

**Abbreviation:** LCEA, lateral central-edge angle.

**Fig. 1:**
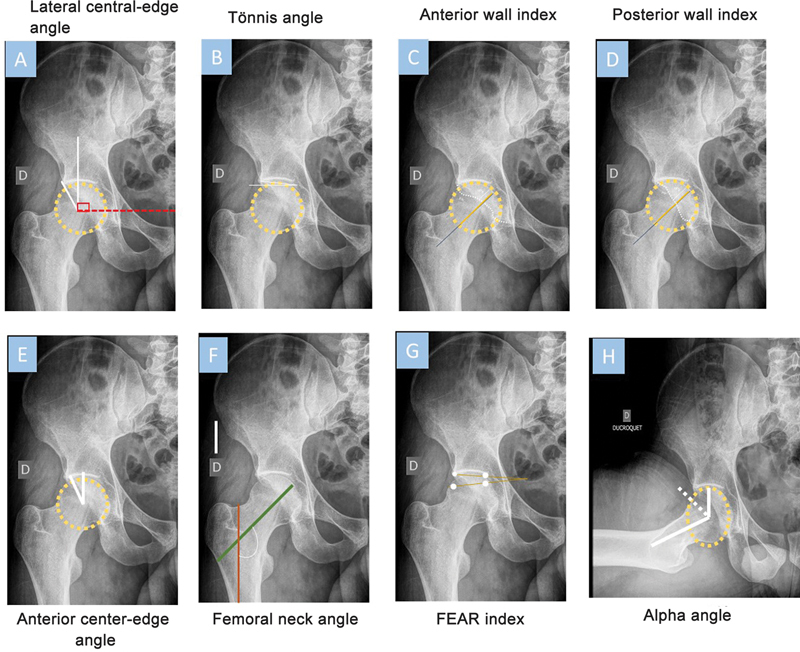
Radiographic measurements to identify borderline hip dysplasia and secondary factors, including hip coverage and instability.

The most frequently used measurement is LCEA ( Fig. 1A ), which defines the superolateral acetabular coverage of the femoral head. Its measurement requires two lines originating at the center of the femoral head. One line extends superiorly and perpendicular to the transverse axis of the pelvis, and the other passes through the lateral edge of the acetabulum. This last line has a more specific definition to intersect the most superolateral point of the sclerotic weight-bearing zone of the acetabulum (sourcil). Studies indicated that LCEA values lower than 20° indicate established DDH. Although some authors consider values from 18 to 25° as borderline dysplasia, the most used and accepted limits in the literature are 20 to 25°. 4

Another measurement to assist borderline DDH diagnosis is the Tönnis angle ( Fig. 1B ). In normal hips, this angle ranges from 0 to 10°, and higher values are common in dysplastic hips. This angle is the caudal-to-cranial inclination of the mid-to-far lateral portion of the acetabular socket with loss of normal lateral acetabular concavity. High Tönnis angle values may indicate borderline DDH, and authors identified a 3-times higher prevalence of these findings in patients with borderline hips compared with healthy subjects. Furthermore, studies showed that patients with hip hypermobility often have higher Tönnis angles, potentially helping surgical decision-making. 17 18

A third measurement of interest is the Sharp angle, measured between a horizontal line drawn at the middle of the bilateral tears (projection of the lower end of the acetabular fossa) and an additional line to the lateral acetabular roof. Studies indicate that Sharp angles between 33 and 38° are normal, while those ranging from 39 to 42° indicate borderline dysplasia, and values above 42° show established dysplasia. 19

A fourth measurement of interest is the iliofemoral line (IFL). Values ranging from 15 to 22% indicate borderline DDH. For measurement, draw a line extending from the apex of the concavity of the lateral femoral neck to the internal cortical margin of the ilium, as well as a horizontal line from the medial to the lateral portion of the femoral head. Then, check how much the vertical line has advanced over the horizontal line as follows: A = size of the horizontal line from the medial edge of the femoral head to the vertical line, and B = size of the horizontal line from the lateral edge of the femoral head up to the vertical line. Then, calculate B/(A + B). In hips with acetabular overcoverage, the IFL is tangential to the femoral head or may be completely lateral to the femoral head. If lateral coverage is lower or the center of the hip presents a superolateral displacement (as in varying degrees of dysplasia), the IFL increasingly intersects the femoral head. 20

The femoroepiphyseal acetabular roof (FEAR) index ( Fig. 1G ) can identify the presence of instability in patients with borderline DDH. To measure it, draw a line over the central portion of the physeal scar and a second line from the medial to the lateral part of the sourcil. An angle inclination in the lateral direction is positive; in this case, values above 5° indicate instability in borderline dysplasia. 4 21

Another angle potentially important in decision-making is the alpha angle ( Fig. 1H ). The alpha angle may guide the surgeon regarding the presence of cam deformities if it is higher than 50°; moreover, studies showed that patients with alpha angles above 78° are at a significantly higher risk of developing advanced-stage osteoarthritis. 22 23 The measurement of this angle occurs in the Dunn and Ducroquet view by drawing a circle around the femoral head and calculating its radius. 24 Mark the point where the distance from the femoral head center exceeds the radius of the femoral head. The alpha angle derives from a line connecting the femoral head center to the point where the distance from this center exceeds the radius and the axis of the femoral neck. Higher alpha angle values may indicate a risk of femoral impingement and the presence of cartilage injuries. 4 23 25

The authors also reported the importance of the femoral neck angle ( Fig. 1F ). To calculate it, draw a line along the center of the femoral shaft and a second line along the center of the femoral neck. These two lines form the femoral neck angle, with reference values ranging from 125 to 135°. 18

Another two useful measurements to assess hip morphology and femoral coverage include the anterior and posterior wall indices. To obtain the anterior wall index (AWI), divide the distance between the center of the femoral head and the anterior wall by the femoral head radius ( Fig. 1C ). Similarly, calculate the posterior wall index (PWI) by dividing the distance from the center of the femoral head to the posterior wall divided by the femoral head radius ( Fig. 1D ). According to Siebenrock et al., 26 AWI values lower than 0.30 or PWI values lower than 0.81 may indicate hip dysplasia.

Another measurement to check hip instability is the anterior center-edge angle ( Fig. 1E ). To calculate it, draw a line from the center of the femoral head to the acetabular sourcil and a line perpendicular to the ground coming from the center of the femoral head. 27 Studies indicate that patients with anterior center-edge angles lower than 20° are more prone to present hip instability. 2

Dornarcher et al. 28 proposed using three indices plus the LCEA to identify different types of borderline dysplasia, as follows: AWI > 0.30 and PWI > 0.80 (laterally dysplastic hip); AWI < 0.30 and PWI < 0.80 (anterolateral dysplastic hip); and AWI > 0.30 and PWI < 0.80 (posterolateral dysplastic hip).

Magnetic resonance imaging exams can identify labral hypertrophy. In patients with no hip conditions, the labrum is around 7.68 mm, whereas in patients with borderline dysplasia, the labrum is closer to 9.44 mm. 4

As for computed tomography, it can provide several measurements to identify the presence of borderline DDH and other factors to assist in decision-making. 15

In axial sections, the acetabular version is the angle between a line perpendicular to the horizontal axis of the posterior acetabulum and a line between the posterior and anterior edges of the acetabulum. This angle usually ranges from 12 to 20°. 29 Studies indicate that values higher than 25° degrees may indicate reduced acetabular coverage and greater instability risk. Low values, indicating acetabular retroversion, may increase the risk of acetabular impact due to excessive anterior acetabular coverage. 15 26 27

The torsion angle of the femur can identify hip instability. Studies demonstrated that the higher this value, the greater the risk of instability. However, multiple methods can measure this angle; as a result, its reference values vary significantly but those ranging from 7 to 24° are normal. 4 30 31 The study by Han et al. 32 demonstrated that subjects without hip dysplasia had lower femoral torsion values than patients with hip dysplasia (21.2 vs. 27.5°, *p* < 0.05). Furthermore, these authors pointed out that the higher the Hartofilakidis grade, the higher the femoral torsion (GI: 24, GII: 29, GIII: 39°).

Finally, in their study, Saks et al. 33 reported that patients with borderline DDH most likely are not a single/homogeneous group, but rather several subgroups with distinct characteristics. Their study investigated the difference between male and female patients with borderline DDH, demonstrating that male patients tend to present larger alpha angles (69.7 vs. 58.1°) than female patients. Male patients also had higher rates of labral injury (62.4 vs. 19.3%) and grade 3 and 4 cartilage injuries (50 vs. 30%). Female patients underwent more capsular plication to treat hip instability (78.5 vs. 45.9%) than males. Furthermore, more females presented painful internal pain requiring fractional iliopsoas stretching than males (60 vs. 32.1%). These data demonstrate that although both groups suffer from the same condition, the differences between radiological parameters and surgical findings are so striking that they could form two different pathological entities. 33

## Treatment of Borderline Developmental Dysplasia of the Hip

There is great debate in the literature about the ideal treatment for patients with borderline DDH since the most used procedures (arthroscopy or periacetabular osteotomy) result in effective outcomes. However, in both techniques, some patients present complications and require a new surgical approach. 3

Swarup et al. 34 studied a retrospective cohort with 33 patients, reporting that the periacetabular osteotomy technique can significantly improve the patient's quality of life and clinical condition in 90% of subjects within one year after the procedure.

Evans et al., 35 in a retrospective analysis of 17 patients with borderline DDH, paired 1:1 with subjects without hip dysplasia, analyzed the impact of hip arthroscopy. These authors reported both groups achieved significant improvement in clinical outcomes, showing that arthroscopy can be an effective treatment for borderline DDH.

Nawabi et al. 36 presented the 2-year clinical outcomes from 55 cases of borderline DDH treated with arthroscopy and compared with a control group (subjects with hips undergoing arthroscopy, but with LCEA > 25° and < 40°). The authors demonstrated that both groups showed significant improvement in the postoperative quality of life questionnaires, with no difference between the improvement of one group over the other, and similar hip movement outcomes. Furthermore, the authors investigated the procedures performed in each group, and the most frequent in patients with borderline DDH was round ligament debridement.

Similarly, Beck et al. 37 performed a retrospective cohort study pairing patients with borderline dysplasia and patients without borderline dysplasia undergoing arthroscopy. The study indicated that both groups showed significant improvement in numerous quality of life questionnaires, significant clinical improvement, and a patient-acceptable symptom state (PASS). Moreover, the study showed that the smaller the alpha angle and the larger the preoperative LCEA, the greater the chance of the patient reaching PASS in 2 years.

Owens et al., 38 in a paired study with a follow-up period of 5 years, reported that high-level athletes with borderline dysplasia undergoing hip arthroscopy presented clinical outcome improvement and returned to sports similarly to athletes with normal hip coverage.

In a study with 56 patients, Domb et al. 39 analyzed the 10-year clinical outcomes of patients with borderline dysplasia who underwent arthroscopy and capsular plication with labral preservation. The authors revealed that these subjects, on average, had a 76% rate of significant clinical improvement in some quality of life questionnaires and a 10-year arthroplasty-free survival rate of 82%. The study also demonstrated that patients with borderline DDH had a similar clinical evolution to patients with normal coverage undergoing the same procedure. However, the survival rate of patients without dysplasia was 92.4%. At last, the authors reported that in the group with borderline DDH, the factors most associated with the need for conversion to arthroplasty were older age and higher body mass index (BMI). 39

Beals et al., 40 in a retrospective cohort study with a minimum follow-up period of 10 years, investigated the clinical outcomes of patients with borderline DDH and FAI. The study demonstrated that patients undergoing arthroscopy showed significant improvement in multiple quality-of-life questionnaires. Additionally, it revealed that the risk factors for conversion to hip arthroplasty were advanced age, presence of advanced chondral lesions, and Tönnis angles higher than 15°.

Several authors investigated the potential risk factors for failed arthroscopies in subjects with borderline dysplasia. Maldonado et al. 41 identified that patients over 35 have a 2.35-fold greater risk of failing capsular plication surgery when compared with patients under 35-years-old. Hatakeyama et al. 42 demonstrated that patients with borderline DDH receiving arthroscopy with labral preservation, capsular plication, and cam osteoplasty had a higher risk of procedure failure if older than 42, with the Shenton line breaking and the Tönnis angles higher than 15°. Maldonado et al. 43 performed a paired study with patients with borderline dysplasia undergoing arthroscopy, revealing that those with an intact/healthy round ligament tended to present better outcomes than patients with round ligament injuries.

A new study by Maldonado et al. 44 investigated the clinical outcomes of patients undergoing revision hip arthroscopy through a second arthroscopy. The authors reported that patients with borderline DDH achieved similar clinical improvements to subjects without dysplasia. Nevertheless, the dysplastic group tended to have a higher risk of requiring a secondary procedure.

McClincy et al., 25 in a study with 49 patients with borderline DDH, identified that they showed and sustained significant clinical improvement two years after undergoing periacetabular osteotomy. Moreover, these authors revealed that the 2-year survival rate without revision in patients undergoing osteotomy was 94%.

Nepple et al., 45 presented a retrospective cohort of 178 patients (186 hips) with borderline DDH who underwent periacetabular osteotomy. Their study demonstrated that these patients showed significant clinical improvement in multiple quality of life questionnaires. Furthermore, the authors revealed that patients who underwent previous arthroscopies had worse clinical outcomes in the last follow-up than those without previous procedures. The authors also investigated the impact of adding concomitant osteoplasties or arthroscopies to periacetabular osteotomy, demonstrating that those who received these additional procedures did not present better clinical outcomes in the last follow-up than subjects who did not receive them. Finally, the authors significantly associated the low LCEA angle correction with treatment failures (failure: 6.6°; and success: 11.1°). 45

Barton et al., 46 in a systematic review, revealed that patients undergoing hip arthroscopy for borderline DDH treatment had a 7.5% rate of revision arthroscopies, approximately 4% of conversion to arthroplasty, and a 13.7% rate of requiring an overall revision.

In another systematic review of medium- and long-term clinical outcomes in patients with borderline DDH, Lee et al. 47 demonstrated that at least 70% of patients achieved important clinical improvement in one or more quality of life questionnaires. Furthermore, the authors reported that the rate of revision arthroscopies ranged from 0 to 7%, a rate of conversions to hip arthroplasties from 0 to 24%, a medium-term revision-free survival rate of 98.2%, and a long-term survival rate of 76.3%. Finally, the study revealed that a Tönnis grade higher than 2, a Tönnis angle higher than 15°, and an Outerbridge grade IV were the main factors associated with the need for conversion to arthroplasty in this group of patients. 47

Murata et al. 48 performed a systematic review to compare the outcomes of periacetabular osteotomy and hip arthroscopy in patients with borderline hip dysplasia. The scarcity of literature comparing these techniques hindered their review. However, both procedures resulted in significant clinical improvements and similar complication rates, ranging from 0 to 22%.

Finally, Andronic et al. 49 compared patients with borderline DDH undergoing periacetabular osteotomy or hip arthroscopy matched by age, gender, and radiological data. The study demonstrated that both groups showed significant improvements in clinical outcomes and a similar rate of minimum important clinical difference (MCID) and PASS. The authors demonstrated that patients undergoing periacetabular osteotomy had a higher risk of future surgery than arthroplasty ones (mainly due to implant reaction). Additionally, there was evidence that three patients from their arthroplasty group required revision and underwent periacetabular osteotomy because of persistent pain, and one required a hip arthroplasty. Meanwhile, a single patient from the periacetabular osteotomy group required arthroscopy to treat intra-articular adhesions. 49

## Final Considerations

Borderline DDH generates much debate in the literature regarding its parameters and best approach methods. Therefore, supplementary tests are essential to identify different patterns of presentation, which can help the surgical decision-making process.

Lastly, there is still no consensus regarding the best approach for borderline DDH, with reviews pointing out the lack of clinical differences between arthroscopies and osteotomies.

## References

[1] Gala L Clohisy J C Beaulé P E Hip Dysplasia in the Young Adult J Bone Joint Surg Am 2016 98 01 63 73 26738905 10.2106/JBJS.O.00109

[2] Kraeutler M J Garabekyan T Pascual-Garrido C Mei-Dan O Hip instability: a review of hip dysplasia and other contributing factors Muscles Ligaments Tendons J 2016 6 03 343 353 28066739 10.11138/mltj/2016.6.3.343 PMC5193524

[3] Kraeutler M J Editorial Commentary: Most Patients With Borderline Hip Dysplasia Do Well After Hip Arthroscopy: Could Instability Be the Problem for Those Who Do Poorly? Arthroscopy 2023 39 02 283 284 36603997 10.1016/j.arthro.2022.10.005

[4] Welton K L Kraeutler M J Garabekyan T Mei-Dan O Radiographic Parameters of Adult Hip Dysplasia Orthop J Sports Med 2023 11 02 23259671231152868 10.1177/23259671231152868 PMC9983115 36874050

[5] Vaudreuil N J McClincy M P Evaluation and Treatment of Borderline Dysplasia: Moving Beyond the Lateral Center Edge Angle Curr Rev Musculoskelet Med 2020 13 01 28 37 32030604 10.1007/s12178-020-09599-y PMC7083976

[6] Grammatopoulos G Pascual-Garrido C Nepple J The Borderline Dysplastic Hip: Arthroscopy or PAO? Orthop J Sports Med 2018 6 07 x

[7] Nepple J J Fowler L M Larson C M Decision-making in the Borderline Hip Sports Med Arthrosc Rev 2021 29 01 15 21 33395225 10.1097/JSA.0000000000000298

[8] Engesæter IØ Laborie L B Lehmann T G Prevalence of radiographic findings associated with hip dysplasia in a population-based cohort of 2081 19-year-old Norwegians Bone Joint J 2013 95-B 02 279 285 23365042 10.1302/0301-620X.95B2.30744

[9] Jacobsen S Sonne-Holm S Søballe K Gebuhr P Lund B The distribution and inter-relationships of radiologic features of osteoarthrosis of the hip. A survey of 4151 subjects of the Copenhagen City Heart Study: the Osteoarthrosis Substudy Osteoarthritis Cartilage 2004 12 09 704 710 15325636 10.1016/j.joca.2004.05.003

[10] Raveendran R Stiller J L Alvarez C Population-based prevalence of multiple radiographically-defined hip morphologies: the Johnston County Osteoarthritis Project Osteoarthritis Cartilage 2018 26 01 54 61 29024801 10.1016/j.joca.2017.10.002 PMC5732866

[11] Freiman S M Schwabe M T Fowler L Clohisy J C Nepple J J Prevalence of Borderline Acetabular Dysplasia in Symptomatic and Asymptomatic Populations: A Systematic Review and Meta-analysis Orthop J Sports Med 2022 10 02 23259671211040455 10.1177/23259671211040455 PMC8832597 35155698

[12] Kapron A L Anderson A E Aoki S K Radiographic prevalence of femoroacetabular impingement in collegiate football players: AAOS Exhibit Selection J Bone Joint Surg Am 2011 93 19 e111, 1–10) 10.2106/JBJS.K.00544 22005872

[13] Kapron A L Peters C L Aoki S K The prevalence of radiographic findings of structural hip deformities in female collegiate athletes Am J Sports Med 2015 43 06 1324 1330 25828079 10.1177/0363546515576908

[14] Harris J D Gerrie B J Varner K E Lintner D M McCulloch P C Radiographic Prevalence of Dysplasia, Cam, and Pincer Deformities in Elite Ballet Am J Sports Med 2016 44 01 20 27 26324404 10.1177/0363546515601996

[15] Kraeutler M J Goodrich J A Ashwell Z R Garabekyan T Jesse M K Mei-Dan O Combined Lateral Osseolabral Coverage Is Normal in Hips With Acetabular Dysplasia Arthroscopy 2019 35 03 800 806 30733038 10.1016/j.arthro.2018.10.133

[16] Multicenter Arthroscopic Study of the Hip (MASH) Study Group Matsuda D K Wolff A B Nho S J Hip Dysplasia: Prevalence, Associated Findings, and Procedures From Large Multicenter Arthroscopy Study Group Arthroscopy 2018 34 02 444 453 29146166 10.1016/j.arthro.2017.08.285

[17] Wong T Y Jesse M K Jensen A Kraeutler M J Coleman C Mei-Dan O Upsloping lateral sourcil: a radiographic finding of hip instability J Hip Preserv Surg 2018 5 04 435 442 30647935 10.1093/jhps/hny042 PMC6328756

[18] McQuivey K S Secretov E Domb B G A Multicenter Study of Radiographic Measures Predicting Failure of Arthroscopy in Borderline Hip Dysplasia: Beware of the Tönnis Angle Am J Sports Med 2020 48 07 1608 1615 32343594 10.1177/0363546520914942

[19] Mannava S Geeslin A G Frangiamore S J Comprehensive Clinical Evaluation of Femoroacetabular Impingement: Part 2, Plain Radiography Arthrosc Tech 2017 6 05 e2003 e2009 29399468 10.1016/j.eats.2017.06.011 PMC5794674

[20] Kraeutler M J Ashwell Z R Garabekyan T The Iliofemoral Line: A Radiographic Sign of Acetabular Dysplasia in the Adult Hip Am J Sports Med 2017 45 11 2493 2500 28609639 10.1177/0363546517708983

[21] Wyatt M Weidner J Pfluger D Beck M The Femoro-Epiphyseal Acetabular Roof (FEAR) Index: A New Measurement Associated With Instability in Borderline Hip Dysplasia? Clin Orthop Relat Res 2017 475 03 861 869 27796801 10.1007/s11999-016-5137-0 PMC5289197

[22] Agricola R Waarsing J H Thomas G E Cam impingement: defining the presence of a cam deformity by the alpha angle: data from the CHECK cohort and Chingford cohort Osteoarthritis Cartilage 2014 22 02 218 225 24269636 10.1016/j.joca.2013.11.007

[23] Nötzli H P Wyss T F Stoecklin C H Schmid M R Treiber K Hodler J The contour of the femoral head-neck junction as a predictor for the risk of anterior impingement J Bone Joint Surg Br 2002 84 04 556 560 12043778 10.1302/0301-620x.84b4.12014

[24] Barton C Salineros M J Rakhra K S Beaulé P E Validity of the alpha angle measurement on plain radiographs in the evaluation of cam-type femoroacetabular impingement Clin Orthop Relat Res 2011 469 02 464 469 20953854 10.1007/s11999-010-1624-x PMC3018186

[25] McClincy M P Wylie J D Kim Y J Millis M B Novais E N Periacetabular Osteotomy Improves Pain and Function in Patients With Lateral Center-edge Angle Between 18° and 25°, but Are These Hips Really Borderline Dysplastic? Clin Orthop Relat Res 2019 477 05 1145 1153 30272611 10.1097/CORR.0000000000000516 PMC6494304

[26] Siebenrock K A Kistler L Schwab J M Büchler L Tannast M The acetabular wall index for assessing anteroposterior femoral head coverage in symptomatic patients Clin Orthop Relat Res 2012 470 12 3355 3360 22798137 10.1007/s11999-012-2477-2 PMC3492620

[27] Clohisy J C Carlisle J C Beaulé P E A systematic approach to the plain radiographic evaluation of the young adult hip J Bone Joint Surg Am 2008 90(Suppl 4, Suppl 4)47 66 10.2106/JBJS.H.00756 PMC2682767 18984718

[28] Dornacher D Lutz B Fuchs M Zippelius T Reichel H Acetabular deficiency in borderline hip dysplasia is underestimated by lateral center edge angle alone Arch Orthop Trauma Surg 2023 143 07 3937 3944 36271941 10.1007/s00402-022-04652-6 PMC10293430

[29] Direito-Santos B França G Nunes J Acetabular retroversion: Diagnosis and treatment EFORT Open Rev 2018 3 11 595 603 30595845 10.1302/2058-5241.3.180015 PMC6275849

[30] Buly R L Sosa B R Poultsides L A Caldwell E Rozbruch S R Femoral Derotation Osteotomy in Adults for Version Abnormalities J Am Acad Orthop Surg 2018 26 19 e416 e425 30106763 10.5435/JAAOS-D-17-00623 PMC6147096

[31] Hartel M J Petersik A Schmidt A Determination of Femoral Neck Angle and Torsion Angle Utilizing a Novel Three-Dimensional Modeling and Analytical Technology Based on CT Datasets PLoS One 2016 11 03 e0149480 26933877 10.1371/journal.pone.0149480 PMC4775021

[32] Han Q Zhang A Wang C Yang K Wang J Application of three-dimensional reconstruction to improve the preoperative measurement accuracy and applicability of femoral neck torsion angle Medicine (Baltimore) 2019 98 45 e17727 31702623 10.1097/MD.0000000000017727 PMC6855581

[33] Saks B R Fox J D Owens J S One Bony Morphology, Two Pathologic Entities: Sex-Based Differences in Patients With Borderline Hip Dysplasia Undergoing Hip Arthroscopy Am J Sports Med 2021 49 14 3906 3914 34694159 10.1177/03635465211043510

[34] Swarup I Zaltz I Robustelli S Sink E Outcomes of periacetabular osteotomy for borderline hip dysplasia in adolescent patients J Hip Preserv Surg 2020 7 02 249 255 33163209 10.1093/jhps/hnaa012 PMC7605771

[35] Evans P T Redmond J M Hammarstedt J E Liu Y Chaharbakhshi E O Domb B G Arthroscopic Treatment of Hip Pain in Adolescent Patients With Borderline Dysplasia of the Hip: Minimum 2-Year Follow-Up Arthroscopy 2017 33 08 1530 1536 28506617 10.1016/j.arthro.2017.03.008

[36] Nawabi D H Degen R M Fields K G Outcomes After Arthroscopic Treatment of Femoroacetabular Impingement for Patients With Borderline Hip Dysplasia Am J Sports Med 2016 44 04 1017 1023 26831630 10.1177/0363546515624682

[37] Beck E C Nwachukwu B U Chahla J Patients With Borderline Hip Dysplasia Achieve Clinically Significant Outcome After Arthroscopic Femoroacetabular Impingement Surgery: A Case-Control Study With Minimum 2-Year Follow-up Am J Sports Med 2019 47 11 2636 2645 31419157 10.1177/0363546519865919

[38] Owens J S Jimenez A E Lee M S Monahan P F Maldonado D R Domb B G High-Level Athletes With Borderline Hip Dysplasia Achieve Favorable Outcomes and Return to Sport Rates Following Primary Hip Arthroscopy: Minimum 5-Year Outcomes Comparison to a Propensity-Matched Control Group Arthroscopy 2023 39 02 271 282 36055477 10.1016/j.arthro.2022.08.023

[39] Domb B G Owens J S Glein R M Jimenez A E Maldonado D R Borderline Dysplasia After Primary Hip Arthroscopy with Capsular Plication and Labral Preservation: Ten-Year Survivorship and Patient-Reported Outcomes J Bone Joint Surg Am 2023 105 09 687 699 37083686 10.2106/JBJS.22.00340

[40] Beals T R Soares R W Briggs K K Day H K Philippon M J Ten-Year Outcomes After Hip Arthroscopy in Patients With Femoroacetabular Impingement and Borderline Dysplasia Am J Sports Med 2022 50 03 739 745 35133204 10.1177/03635465211068109

[41] Maldonado D R Perets I Mu B H Arthroscopic Capsular Plication in Patients With Labral Tears and Borderline Dysplasia of the Hip: Analysis of Risk Factors for Failure Am J Sports Med 2018 46 14 3446 3453 30419179 10.1177/0363546518808033

[42] Hatakeyama A Utsunomiya H Nishikino S Predictors of Poor Clinical Outcome After Arthroscopic Labral Preservation, Capsular Plication, and Cam Osteoplasty in the Setting of Borderline Hip Dysplasia Am J Sports Med 2018 46 01 135 143 28992426 10.1177/0363546517730583

[43] Maldonado D R Chen S L Walker-Santiago R An Intact Ligamentum Teres Predicts a Superior Prognosis in Patients With Borderline Dysplasia: A Matched-Pair Controlled Study With Minimum 5-Year Outcomes After Hip Arthroscopic Surgery Am J Sports Med 2020 48 03 673 681 32017862 10.1177/0363546519898716

[44] Maldonado D R Kyin C Shapira J Revision Hip Arthroscopy in the Borderline Dysplastic Population: Reporting Outcomes With Minimum 2-Year Follow-up, With a Subanalysis Against a Propensity-Matched Nondysplastic Control Group Am J Sports Med 2021 49 01 66 75 33216619 10.1177/0363546520969878

[45] Nepple J J Parilla F W Pashos G E Clohisy J C Outcomes of Periacetabular Osteotomy for Borderline Acetabular Dysplasia J Bone Joint Surg Am 2023 105 02 137 144 36651889 10.2106/JBJS.22.00491

[46] Barton C Scott E Khazi Z M Willey M Westermann R Outcomes of Surgical Management of Borderline Hip Dysplasia: A Systematic Review Iowa Orthop J 2019 39 02 40 48 32577106 PMC7047291

[47] Lee M S Owens J S Fong S Mid- and Long-Term Outcomes Are Favorable for Patients With Borderline Dysplasia Undergoing Primary Hip Arthroscopy: A Systematic Review Arthroscopy 2023 39 04 1060 1073 36596369 10.1016/j.arthro.2022.12.030

[48] Murata Y Fukase N Martin M Comparison Between Hip Arthroscopic Surgery and Periacetabular Osteotomy for the Treatment of Patients With Borderline Developmental Dysplasia of the Hip: A Systematic Review Orthop J Sports Med 2021 9 05 23259671211007401 10.1177/23259671211007401 PMC8113971 33997083

[49] Andronic O Chaharbakhshi E O Zingg P O No Difference in Patient-Reported Outcomes for Periacetabular Osteotomy and Hip Arthroscopy With Capsular Plication in the Setting of Borderline Hip Dysplasia: A Propensity-Matched Multicenter Study With Minimum 5-Year Follow-up Arthroscopy 2024 40 03 754 762 37422025 10.1016/j.arthro.2023.06.045

