# Supplementary material for: Painful Borderline Acetabular Dysplasia: What's New?
Source: Rev Bras Ortop (Sao Paulo). 2025 Apr 2;60(1):s00441790212. doi: 10.1055/s-0044-1790212 (PMC11964716; doi:10.1055/s-0044-1790212)
Supplement: Related texts

# Research article: Displasia acetabular limítrofe dolorosa: O que há de novo?

**Keywords:** articulação do quadril, diagnóstico por imagem, impacto femoroacetabular, luxação do quadril, tomada de decisões

## Abstract

A displasia do desenvolvimento do quadril (DDQ), é uma patologia estática-dinâmica complexa que conduz à instabilidade articular crônica e à artrose. Já a displasia acetabular *borderline* (limítrofe) refere-se a padrões levemente anormais na forma e na cobertura acetabular que não estão dentro dos valores para serem considerados displásicos. Entretanto, tais padrões podem predispor a disfunção mecânica e instabilidade quadril. O diagnóstico e o tratamento continuam sendo um tópico controverso na preservação do quadril, com pouca literatura comparativa atual para orientar o diagnóstico preciso e a tomada de decisão do tratamento. Historicamente o diagnóstico da DDQ limítrofe foi baseado em avaliações da anatomia acetabular na radiografia pélvica anteroposterior, mais comumente o ângulo da borda central lateral (LCEA), com valores normais entre 20 e 25°, ou, em alguns estudos mais recentes, 18 a 25°. Quanto a tomada de decisão de tratamento em casos cirúrgicos, há grande debate entre a utilização artroscopia isolada do quadril ou osteotomia periacetabular, tendo em vista que diagnóstico mecânico fundamental (instabilidade versus impacto femoroacetabular) pode ser difícil de determinar nos casos de DDQ limítrofe. Assim, para uma tomada de decisão cirúrgica efetiva, a avaliação de características adicionais da anatomia óssea, instabilidade e características dos pacientes é fundamental.

## Introdução

A displasia do desenvolvimento do quadril (DDQ) é uma condição musculoesquelética importante. Embora cerca de 80% dos casos já estejam presentes desde o nascimento, muitas vezes tais alterações permanecem sem diagnóstico apropriado. Apesar de a maioria dos casos de DDQ serem assintomáticos, essa condição pode levar a alterações biomecânicas na região do quadril, que podem predispor os indivíduos ao desenvolvimento de quadros de osteoartrite do quadril. 1

A classificação desta condição é, em geral, baseada nos valores de ângulo centro-borda lateral (LCEA) de Wiberg menores que 20°. 2 Porém, estudos demonstram que quadris com valores entre 20 e 25° também apresentam sinais de displasia, no entanto, nestes casos as apresentações imaginológicas, biomecânicas e clínicas, tendem a ser diferentes da DDQ estabelecida. Deste modo, tais casos passaram a ser denominados DDQ limítrofe.

O uso do termo “limítrofe” em si é um tanto controverso, com uma definição variável na literatura. É importante reconhecer que esse termo não indica que a deficiência acetabular não é importante, mas sim é utilizado para reconhecer que o diagnóstico primário desses quadris pode se enquadrar em qualquer lado do espectro de instabilidade e impacto estabelecidos na literatura. 3 4

Um dos grandes dilemas relacionados à DDQ limítrofe é que, diferentemente da estabelecida, alguns dos pacientes apresentam instabilidade estrutural (similar a casos de displasia estabelecida), ao passo que outros pacientes apresentam impacto femoroacetabular (IFA) e microinstabilidade. 5 6 Assim, a tomada de decisões cirúrgicas, quando necessárias, em quadris com displasia acetabular limítrofe é desafiadora devido à ausência de padrões clínicos para diferenciar quadris com instabilidade estrutural significativa daqueles com IFA e microinstabilidade/ausência de instabilidade. 7

Deste modo, dada a relativa escassez e heterogeneidade da literatura quanto a DDQ limítrofe, o objetivo deste trabalho é realizar uma revisão da literatura para identificar os conceitos mais atuais quanto ao diagnóstico, classificação, tratamento e desfechos clínicos referentes à doença.

## Materiais e Métodos

## Revisão Narrativa da Literatura

Para auxiliar no processo de busca e aquisição dos artigos foi realizada uma ampla revisão da literatura nas seguintes bases de dados: Medline (PubMed), OVID, Google Scholar e Scielo. Para cada uma das bases foi criada uma estratégia de busca para identificação de artigos de interesse. Exemplos dos mecanismos de busca são citados a seguir: *borderline hip dysplasia* AND *imaging diagnostic* OR *diagnostic* OR *x-ray* OR *magnetic resonance* OR *computer tomography* ou *borderline hip dysplasia* AND *treatment* OR *surgical treatment* OR *hip arthroscopy* OR *periacetabular osteotomy* .

Após identificação dos artigos, foram excluídos aqueles que não mencionavam o tema displasia do quadril, artigos em outras línguas que não português ou inglês, e artigos que não puderam ser acessados pelos autores.

## Epidemiologia

Por se tratar de uma condição limítrofe e com muitos debates quanto a sua classificação, a real prevalência da DDQ limítrofe ainda não é completamente compreendida, tendo em vista que muitos estudos classificam quadris com a condição como normais ou não têm seus valores reportados. Múltiplos parâmetros podem ser utilizados para identificação, entre eles ângulos de LCEA entre 20 e 25°, ângulos de Sharp maiores que 45° e o ângulo de Tönnis > 10°. 5 8

## Epidemiologia – População Geral

Jacobsen et al., 9 relata em estudo com 3.859 pacientes assintomáticos, que a prevalência de quadris classificados com DDQ limítrofe foi de 19.2%, ao passo que apenas 3,4% dos pacientes apresentaram DDQ estabelecida.

Ademais, Engesæter et al., 8 em estudo prospectivo com 2.072 indivíduos de 19 anos, identificou que 16,7% dos pacientes apresentam sinais de DDQ limítrofe e que 3,3% apresentavam sinais de displasia estabelecida. Mais recentemente, um estudo norte-americano com 2.596 pacientes derivados de uma base de dados coletada entre 1990 e 1997 para detecção da prevalência de osteoartrite no condado de Johnston, Carolina do Norte, apontou que DDQ limítrofe envolveu aproximadamente 18,8% dos casos. 10

Por fim, Freiman et al. 11 realizaram uma metanálise para identificar a prevalência geral de DDQ limítrofe. Os autores verificam uma presença de 6,7% entre os pacientes assintomáticos. Ademais, os autores apontam que na população geral assintomática a DDQ limítrofe ocorre 3,5 vezes mais que a clássica. Entretanto, em populações sintomáticas a diferença reduz para 1,3 vezes.

## Epidemiologia – População de Atletas Profissionais

Além do mais, Kapron et al., 12 em uma população com 67 jogadores de futebol americano assintomáticos, verificaram a presença de 19,4% DDQ limítrofes. Em estudo do mesmo grupo, desta vez com grupo de 63 atletas femininas assintomáticas com média de 19 anos de idade, apontou que 46% destas apresentavam quadril limítrofe e 20% DDQ estabelecida. 13 Por fim, Harris et al. 14 investigaram um grupo de 47 profissionais de uma companhia de ballet e verificou-se que 51% dos pacientes tinham DDQ limítrofe.

## Epidemiologia – População sintomática

Quanto a prevalência de DDQ limítrofe em pacientes com dores no quadril, Kraeutler et al., 15 2019, reportam 341 indivíduos que a presença de displasia limítrofe era de 14% entre os pacientes. Já Matsuda et al., 16 investigando 1.053 pacientes na fila para receberem artroscopia do quadril, identificou uma prevalência de 12,6%.

## Diagnóstico por Imagem

O diagnóstico da DDQ limítrofe só pode ser concluído com o auxílio de exames de imagem e a mensuração de ângulos associados às alterações causadas pela displasia. A Tabela 1 apresenta a comparação entre os valores de quadris com displasia limítrofe e estabelecida.

**Tabela 1:** Parâmetros associados a diagnósticos de displasia limítrofe ou de displasia franca

Parâmetro	Displasia limítrofe	Displasia franca
LCEA	20–25°	< 20°
Linha illiofemoral	15–22%	> 22%
Ângulo centro borda anterior	20–25°	< 20°
Ângulo de Tönnis	> 13°	> 13°
Ângulo de Sharp	39–42°	> 42°

Abreviações: LCEA, ângulo centro-borda lateral.

A medida mais frequentemente utilizada é o LCEA ( Fig. 1A ), que define a cobertura acetabular súperolateral da cabeça do fêmur, e é medido por meio de duas linhas que se originam no centro da cabeça femoral. Uma linha estende-se superiormente e perpendicularmente ao eixo transverso da pelve e a outra passa pela borda lateral do acetábulo. Esta última linha foi mais especificamente definida para cruzar o ponto mais superolateral da zona de sustentação de peso esclerótica do acetábulo (sourcil). Estudos apontam que valores de LCEA menores que 20° são indicativos de DDQ estabelecida. Alguns autores chegam a considerar valores entre 18 e 25° como displasias limítrofes, no entanto, os limites mais utilizados e aceitos na literatura são de 20 a 25°. 4

**Fig. 1:** Apresentação das mensurações em raio-X que podem ser utilizadas na identificação da displasia de quadril borderline e na identificação de fatores secundários, como cobertura do quadril, presença de instabilidade, entre outros.

Outra medida que pode auxiliar no diagnóstico da DDQ limítrofe é o ângulo de Tönnis ( Fig. 1B ), que possui valores normativos entre 0 e 10°, com valores maiores geralmente sendo encontrados em quadris displásicos. Este ângulo é definido como uma inclinação caudal para cranial da porção média a lateral distante do soquete acetabular com perda da concavidade acetabular lateral normal. Valores elevados de ângulo de Tönnis podem indicar a presença de DDQ limítrofe, com autores identificando uma prevalência 3 vezes maior destes achados em pacientes com quadril limítrofe em relação a pacientes saudáveis. Ademais, estudos apontam que pacientes com hipermobilidade do quadril tendem a apresentar maiores valores de ângulo de Tönnis, o que pode auxiliar na tomada de decisão cirúrgica. 17 18

A terceira medida de interesse é o ângulo de Sharp que é medido por uma linha horizontal traçada entre as lágrimas bilaterais (projeção da extremidade inferior da fossa acetabular) e uma linha adicional até o teto acetabular lateral. Estudos apontam que ângulos de Sharp entre 33 e 38° são considerados normativos, valores entre 39 e 42°, definidos como indicadores de displasia limítrofe; e valores acima de 42° sendo considerados como quadris com displasia estabelecida. 19

Outra medida de interesse é a linha iliofemoral (LIF), na qual valores entre 15 e 22% são indicativos de DDQ limítrofe. Este parâmetro é medido com uma linha que se estende desde o ápice da concavidade do colo femoral lateral até a margem cortical interna do ílio; adicionalmente uma linha horizontal da porção medial à porção lateral da cabeça do fêmur é desenhada. Então, verifica-se o quanto a linha vertical avançou sobre a linha horizontal da seguinte forma: A = tamanho da linha horizontal do bordo medial da cabeça do fêmur até a linha vertical; e B = tamanho da linha horizontal do bordo lateral da cabeça do fêmur até a linha vertical. Por fim realiza-se a seguinte conta B/(A + B). Em quadris com supercobertura acetabular, a LIF é tangente à cabeça do fêmur ou pode até mesmo estar completamente lateral à cabeça. Se a cobertura lateral é reduzida ou o centro do quadril é deslocado de maneira superolateral (como em graus variados de displasia), a LIF intersecta cada vez mais a cabeça do fêmur. 20

Já, o índice femoroepifiseal do teto do acetábulo (do inglês, FEAR) pode ser utilizado para identificar a presença de instabilidade em pacientes com DDQ limítrofe ( Fig. 1G ). A medida é composta por uma linha sobre a porção central da cicatriz fiseal, e uma segunda linha que é feita da parte medial à parte lateral do sourcil, caso a inclinação do ângulo seja na direção lateral este é considerado positivo; neste caso, valores acima de 5° são indicativos de instabilidade em displasia limítrofe. 4 21

Outro ângulo que pode ser importante na tomada de decisão é o ângulo alfa ( Fig. 1H ) que pode orientar o cirurgião quanto a presença de deformidades do tipo *cam* , caso seus valores estejam acima de 50°; ademais, estudos apontam que pacientes com ângulos alfa maiores que 78° apresentam risco significativamente maior de desenvolverem osteoartrite de estágio avançado. 22 23 Este ângulo é medido na incidência de Dunn e Ducroquet, por um círculo ao redor da cabeça do fêmur, de onde raio da cabeça do fêmur será derivado. 24 O ponto onde a distância a partir do centro da cabeça excede o raio da cabeça do fêmur é marcado. O ângulo alfa é o ângulo formado por uma linha que conecta o centro da cabeça do fêmur ao ponto onde a distância a partir do centro da cabeça excede o raio da cabeça e o eixo do colo do fêmur. Valores maiores de ângulo alfa podem ser indicativos de risco de impacto femoral e da presença de lesões de cartilagem. 4 23 25

Autores também reportam a importância do ângulo colo do fêmur ( Fig. 1F ), onde uma linha é desenhada ao longo do centro do eixo femoral e uma segunda linha é desenhada ao longo do centro do colo femoral. O ângulo do colo do fêmur é o ângulo formado entre essas duas linhas sendo que os valores considerados normativos variam entre 125 e 135°. 18

Outras duas medidas muito úteis na avaliação da morfologia do quadril e cobertura femoral, incluem o índice da parede anterior e índice da parede posterior. O índice da parede anterior (AWI, do inglês) é obtido com a divisão da distância ente o centro da cabeça do fêmur à parede anterior e o raio da cabeça do fêmur ( Fig. 1C ). Similarmente, o índice da parede posterior (PWI, do inglês) pode ser calculado pela divisão da distância do centro da cabeça do fêmur até a parede posterior divido pelo raio da cabeça do fêmur ( Fig. 1D ). De acordo com Siebenrock et al. 26 valores de AWI menores que 0,30 ou de PWI menores 0,81 podem ser indicativos de displasia de quadril.

Outra medida que também pode ser utilizada para verificar a presença de instabilidade nos quadris é o ângulo centro-borda anterior ( Fig. 1E ), que é formado por uma linha que vem do centro da cabeça do fêmur até a fonte acetabular e uma linha perpendicular ao solo vinda do centro da cabeça do fêmur. 27 Estudos apontam que pacientes com valores de ACE menores que 20° estão mais sujeitos a apresentarem instabilidade no quadril. 2

Dornarcher et al. 28 propõe a utilização de três índices em conjunto com o LCEA para a identificação de diferentes tipos de displasia limítrofes, sendo eles: AWI > 0,30 e PWI > 0,80 (quadril displásico lateralmente); AWI < 0,30 e PWI < 0,80 (quadril displásico ântero-lateral); e AWI > 0,30 e PWI < 0,80 (quadril displásico posterolateral).

Exames de ressonância podem ser utilizados para identificação da hipertrofia labral, que em pacientes sem patologias do quadril gira em torno de 7,68 mm, já em pacientes com displasia limítrofe tende a apresentar valores próximos a 9,44 mm. 4

Quanto a exames de tomografia computadorizada, diversas mensurações podem ser feitas para identificação da presença de DDQ-limítrofe e outros fatores para auxiliar na tomada de decisão. 15

A versão acetabular, medida em cortes axiais, é o ângulo entre uma linha perpendicular ao eixo horizontal do acetábulo posterior e uma linha entre a borda posterior e anterior do acetábulo, esse ângulo tem seus valores normativos entre 12 e 20°. 29 Estudos apontam que valores maiores que 25° graus podem ser indicativos de cobertura acetabular reduzida e maiores riscos de presença de instabilidade. Já valores muito reduzidos, indicando uma retroversão acetabular, podem aumentar o risco de impacto acetabular, devido à presença de cobertura acetabular anterior excessiva. 15 26 27

O ângulo de torsão do fêmur também pode ser utilizado para identificar a presença de instabilidade nos quadris, com estudos demonstrando que quanto maior valor deste maior o risco da presença de instabilidade. No entanto, múltiplos métodos são descritos para medir esse ângulo, de forma que os seus valores de referência variem significativamente, com valores entre 7 e 24° sendo considerados normais. 4 30 31 No estudo realizado por Han et al., 32 os autores demonstraram que pacientes sem displasia do quadril apresentavam menores valores de torsão femoral comparados a pacientes com displasia de quadril (21,2° vs. 27,5°, *p* < 0.05). Ademais, os autores apontam que quanto maior o grau de Hartofilakidis, maiores os valores de torsão femoral (GI: 24, GII: 29, GIII: 39°).

Por fim, Saks et al., 33 em seu estudo, demonstram que os pacientes com DDQ-limítrofe muito provavelmente não compõe um grupo único/homogêneo, mas sim são formados por diversos subgrupos com características distintas. Em seu estudo os pesquisadores investigaram a diferença entre os pacientes do sexo masculino e feminino portadores de DDQ limítrofe, demonstrando que pacientes do sexo masculino tendem a apresentar ângulos alfa maiores quando comparados a pacientes do sexo feminino (69,7 vs. 58.1°). Pacientes do sexo masculino também apresentaram maior taxa de lesão labral (62,4 vs. 19,3%) e maiores taxas de lesão de cartilagem grau 3 e 4 (50 vs. 30%). Já pacientes do sexo feminino receberam maior quantidade de plicatura capsular para tratamento de instabilidade do quadril 78,5 contra 45,9% em pacientes do sexo masculino. Ademais, pacientes do sexo feminino apresentaram dor interna dolorosa que requeria alongamento fracional do iliopsoas em maior quantidade do que pacientes do sexo masculino (60 vs. 32,1%). Esses dados demonstram que, apesar dos dois grupos sofrerem da mesma patologia, as diferenças entre os parâmetros radiológicos e achados cirúrgicos é tão marcante que poderiam compor duas entidades patológicas diferentes. 33

## Tratamento da Displasia Desenvolvimental do Quadril Limítrofe

Há grande debate na literatura sobre qual o tratamento ideal para pacientes com DDQ limítrofe, tendo em vista que os procedimentos mais utilizados (artroscopia ou osteotomia periacetabular) apresentam resultados efetivos. No entanto, em ambas as técnicas, alguns pacientes apresentaram complicações e precisaram de uma reabordagem cirúrgica. 3

Swarup et al. 34 reportam, em uma coorte retrospectiva com 33 pacientes, que a utilização da técnica de osteotomia Peri acetabular é capaz de promover melhora clínica significativa na qualidade de vida pacientes e que o procedimento foi capaz de promover melhora clínica significativa em 90% dos pacientes em até um após o procedimento.

Evans et al., 35 em uma análise retrospectiva de 17 pacientes com DDQ limítrofe pareados 1:1 com pacientes sem displasia do quadril, analisaram o impacto da utilização de artroscopia de quadril neste grupo. Os pesquisadores demonstram que ambos os grupos obtiveram melhora clínica significativa dos desfechos clínicos, demonstrando que a artroscopia pode ser um tratamento eficaz para quadros de DDQ limítrofe.

Nawabi et al. 36 apresentam os resultados clínicos de 2 anos de 55 quadris com DDQ limítrofe, tratados com artroscopia e comparados a um grupo controle (quadris que receberam artroscopia, mas tinham LCEA > 25° e < 40°). Os autores demonstraram que ambos os grupos apresentam melhora significativa nos questionários de qualidade de vida pós-operatórios e sem diferença entre a melhora de um grupo e outro, resultados similares aos obtidos nos desfechos de movimento do quadril. Ademais, os autores investigaram os procedimentos que foram realizados em cada um dos grupos, com o de desbridamento do ligamento redondo sendo o procedimento realizado com maior frequência em pacientes com DDQ limítrofe.

De maneira similar, Beck et al., 37 2019, realizaram um estudo de coorte retrospectivo com pareamento entre pacientes com displasia limítrofe e pacientes sem displasia limítrofe recebendo artroscopia. O estudo aponta que ambos os grupos apresentam melhora significativa em diversos questionários de qualidade de vida, além de serem capazes de atingirem melhora clínica significativa e atingirem estado aceitável de sintomas (PASS, do inglês). Ademais, o trabalho aponta que quanto menor o ângulo alfa e quanto maior o LCEA pré-operatórios, maior a chance de o paciente atingir um estado aceitável de sintomas em 2 anos.

Owens et al., 38 reporta que atletas de alto nível com displasia limítrofe submetidos à artroscopia do quadril, em estudo pareado com 5 anos de acompanhamento, apresentam resultados de retorno ao esporte e de melhora nos desfechos clínicos similares à atletas com cobertura normal dos quadris.

Em estudo com 56 pacientes, Domb et al., 39 2023, analisou os desfechos clínicos de 10 anos de pacientes com displasia limítrofe submetidos à artroscopia e plicatura capsular com preservação labral. Os autores apontam que os pacientes apresentam em média uma taxa 76% de atingimento de melhora clínica significativa em algum dos questionários de qualidade de vida e uma taxa de sobrevivência livre aos 10 anos de 82% para artroplastia. O estudo também demonstrou que pacientes com DDQ limítrofe apresentaram evolução clínica similar a pacientes com cobertura normal recebendo o mesmo procedimento, no entanto os pacientes sem displasia apresentaram uma taxa de sobrevivência de 92.4%. Por fim, os autores demonstram que, no grupo com DDQ limítrofe, os fatores mais associados a necessidade de conversão para artroplastias nestes casos foram idade mais avançada e índice de massa corporal (IMC) mais elevado. 39

Beals et al., 40 em um estudo de coorte retrospectiva com acompanhamento mínimo de 10 anos, investigou os desfechos clínicos de pacientes com DDQ limítrofe e impacto femoroacetabular. O estudo demonstrou que os pacientes submetidos a artroscopia apresentaram melhora significativa em múltiplos questionários de qualidade de vida. Além disso, revelou-se que os fatores de risco para conversão para artroplastia de quadril foram, idade avançada, presença de lesões condrais avançadas e ângulos de Tönnis maiores que 15°.

Diversos autores investigam os possíveis fatores de risco para falhas nas artroscopias no cenário de displasia limítrofe. Maldonado et al., 41 2018, identificou pacientes acima de 35 anos como tendo um risco 2.35 vezes maior de falha na cirurgia de plicatura capsular, quando comparado com pacientes com menos de 35 anos. Hatakeyama et al., 42 2018, demonstrou que pacientes com DDQ-limítrofe recebendo artroscopia com preservação labral, plicatura capsular e osteoplastia do *cam* , apresentavam maiores riscos de falha no procedimento quando com idade > 42 anos, quebra da linha de Shenton e ângulo de Tönnis maior que 15°. Já Maldonado et al., 43 2020, aponta em estudo pareado que pacientes com displasia limítrofe submetidos a artroscopia e que tenham um ligamento redondo intacto/saudável tendem a apresentar melhores resultados que pacientes com lesões no ligamento redondo.

Em um novo estudo de Maldonado et al., 44 2021, investigou os desfechos clínicos de pacientes submetidos à revisão de artroscopia do quadril por meio de uma segunda artroscopia. Os autores apontam que no grupo de pacientes com DDQ-limítrofe os pacientes atingiram melhoras clínicas similares aos pacientes sem displasia, no entanto o grupo displásico apresentou uma tendência à maior risco de necessitar um procedimento secundário.

McClincy et al., 25 em estudo com 49 pacientes com DDQ-limítrofe, identificaram que este grupo de pacientes apresentou e manteve melhora clínica significativa 2 anos após receber osteotomia periacetabular. Ademias os autores apontam que a taxa de sobrevivência sem revisão de 2 anos de pacientes submetidos à osteotomia foi de 94%.

Nepple et al. 45 apresentam uma coorte retrospectiva de 178 pacientes (186 quadris) com DDQ limítrofe que receberam osteotomia periacetabular (OP). É demonstrado no estudo que pacientes recebendo OP apresentam melhora clínica significativa em múltiplos questionários de qualidade de vida. Ademais, os autores demonstram que pacientes submetidos a artroscopias prévias, apresentavam piores desfechos clínicos no último período de acompanhamento, compara do àqueles sem procedimentos prévios. Os autores também investigam impacto da adição de osteoplastias ou artroscopias concomitantes à OP, demonstrando que os pacientes que receberam tais procedimentos adicionais, não apresentaram melhores desfechos clínicos no último período de acompanhamento, quando comparados com os pacientes que não receberam os procedimentos. Por fim os autores apontam que o fator que está significativamente associado às falhas no tratamento, é baixa correção de ângulo de LCEA (falha: 6,6 e sucesso: 11,1°). 45

Barton et al., 46 em revisão sistemática, demonstrou que pacientes recebendo artroscopia de quadril para tratamento de DDQ limítrofe apresentaram uma taxa de 7,5% de necessidade de uma artroscopia de revisão, cerca de 4% com conversão para artroplastia e 13,7% de necessidade de revisão em geral.

Em outra revisão sistemática dos desfechos clínicos de médio e longo prazo de pacientes com DDQ limítrofe, Lee et al. 47 demonstram que ao menos 70% dos pacientes atingiram melhora clínica importante em um ou mais questionários de qualidade de vida. Ademais, os autores apontam que a taxa de artroscopias de revisão variou de 0 a 7% e a taxa de conversões para artroplastias de quadril variou de 0 a 24%, sendo que a taxa de sobrevivência sem revisão à médio prazo foi de 98,2% e a taxa de sobrevivência à longo prazo foi de 76,3%. Por fim, o estudo aponta que um grau de Tönnis maior que 2, ângulo de Tönnis maior que 15° e grau IV de Outerbridge são os fatores mais associados à necessidade de conversão para artroplastia nesse grupo de pacientes. 47

Murata et al., 48 2021, realizou uma revisão sistemática para comparar os desfechos entre a realização de osteotomia periacetabular e artroscopia de quadril em pacientes com displasia de quadril limítrofe. Devido à pouca literatura realizando a comparação entre as técnicas não foi possível realizar comparação entre os grupos. Entretanto, ambos os grupos apresentaram melhora clínica significativa e taxas similares de complicações entre os estudos, na faixa de 0 a 22%.

Por fim, Andronic et al. 49 realizou a comparação entre pacientes com DDQ limítrofe recebendo OP ou artroscopia do quadril pareados segundo idade, gênero e dados radiológicos. O estudo demonstra que ambos os grupos apresentaram melhora clínica significativa nos desfechos clínicos e uma taxa similar de diferença clínica significativa (MCID, do inglês) e PASS. Os autores demonstraram que pacientes recebendo OP apresentavam maior risco de cirurgia futura em relação àqueles recebendo artroplastia (principalmente por reação ao implante). Além disso, é evidenciado que dos pacientes do grupo artroplastia, três necessitaram revisão e foram submetidos à osteotomia periacetabular devido à dor persistente, já um paciente do grupo OP necessitou da realização de uma artroscopia para tratamento de adesões intra-articulares. Por fim, um paciente do grupo artroscopia necessitou uma artroplastia de quadril. 49

## Considerações Finais

A DDQ limítrofe é uma condição que gera muito debate na literatura, quanto a sua parametrização e melhores métodos para abordagem. Deste modo, exames complementares são essenciais para auxiliar na identificação de diferentes padrões de apresentação, podendo auxiliar na tomada de decisões cirúrgicas.

Por fim, ainda não há consenso quanto a melhor abordagem para DDQ limítrofe, com revisões apontando que não há diferenças clínicas entre a utilização de artroscopias e osteotomias.
